# A metastable polymorphic form of the anti­fungal anilino­pyrimidine active pyrimethanil

**DOI:** 10.1107/S2056989017007563

**Published:** 2017-05-26

**Authors:** Alex R. Eberlin, Christopher S. Frampton

**Affiliations:** a Johnson Matthey, Pharmorphix, 250 Cambridge Science Park, Milton Road, Cambridge CB4 0WE, England; b Wolfson Centre for Materials Processing, Brunel University London, Kingston Lane, Uxbridge UB8 3PH, England

**Keywords:** crystal structure, polymorphism, pyrimethanil, hydrogen bonding

## Abstract

A second metastable form of the title compound, C_12_H_13_N_3_ (systematic name: 4,6-dimethyl-*N*-phenyl­pyrimidin-2-amine), was isolated from an attempted co-crystallization experiment with *meso*-erythriol in dimethyl sulfoxide (DMSO). The crystals of form 2 at 120 K are monoclinic, space group *P*2_1_/*n* with *Z*′ = 4 compared to the previously reported triclinic form with *Z*′ = 2 [Sun *et al.* (2011 ▸). *Acta Chim. Sin.* **69**, 1909–1914]. The four independent mol­ecules in the asymmetric unit form two discrete dimeric units through a concerted pair of N—H⋯N hydrogen bonds with a graph-set notation of *R*_2_^2^(8). The origin of the polymorphic behaviour is revealed in that the conformation of each dimer present in the asymmetric unit of the structure is unique and determined by the rotation of the second mol­ecule in the dimer with respect to the first.

## Abstract 2

A second metastable polymorphic form of the anti­fungal anilino­pyrimidine active pyrimethanil was isolated from an attempted co-crystallization experiment with *meso*-erythriol in dimethyl sulfoxide (DMSO). The origin of the polymorphic behaviour is revealed in that the conformation of each dimer present in the asymmetric unit of the structure is unique and determined by the rotation of the second mol­ecule in the dimer with respect to the first.

## Chemical context   

(4,6-Dimethyl-pyrimidin-2-yl)-phenyl-amine, pyrimethanil (**1**) is a broad spectrum systemic fungicide from the anilino­pyrimidine class of agents, which also include cyprodinil and mepanipyrim. It was discovered in 1987 (Buhmann *et al.*, 1988 ▸) and is marketed under the trade name SCALA^®^. Anilino­pyrimidines are used extensively for protection against leaf moulds and other fungi. In a recent paper (Sun *et al.*, 2011 ▸), the synthesis and electronic properties of pyrimethanil were presented, including a discussion on the atomic charges, total energy and frontier orbital energy. As part of this wider study, the crystal structure of pyrimethanil was determined at 295 K and used as an initial starting model in the structural optimization process. The structure was triclinic, space group *P* 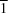, with *Z′* = 2, with two independent mol­ecules in the asymmetric unit. The two independent mol­ecules form a dimeric structural unit through a concerted pair of N—H⋯N hydrogen bonds with a graph-set notation of 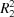
(8). We have recently been investigating the co-crystallization behaviour of pyrimethanil in an attempt to modify the physicochemical properties of the bulk solid material to improve its overall performance. During the course of one of the co-crystallization screens, the crystal structure of a second polymorphic crystal form of pyrimethanil was determined on a crystal that was isolated from the reaction product of an attempted co-crystallization experiment with *meso*-erythriol in di­methyl­sulfoxide (DMSO). In this communication, we report the single crystal X-ray structure of this second, metastable, monoclinic polymorphic form of pyrimethanil at 120 K.
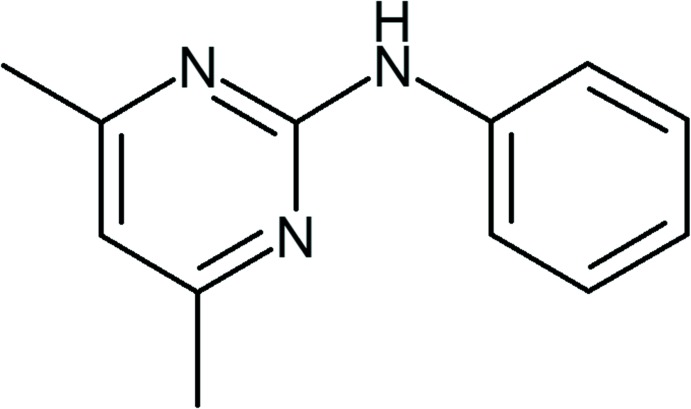


## Structural commentary   

**Figure 1:**
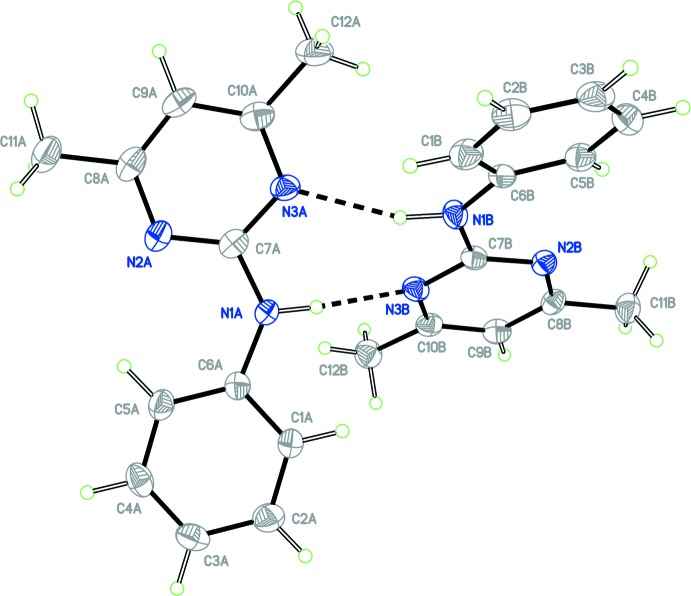
View of the *A*–*B* dimer of the asymmetric unit with atom labelling. Ellipsoids are drawn at the 50% probability level. The inter­molecular N—H⋯N hydrogen bonds are shown as dashed lines.

The crystal structure of form 2 of pyrimethanil is monoclinic, space group *P*2_1_/*n* with four independent mol­ecules of pyrimethanil in the asymmetric unit, (*Z*′ = 4). For clarity, the independent mol­ecules are labelled with suffixes *A*, *B*, *C* and *D*. The four independent mol­ecules arrange themselves into two dimeric units *A*–*B* and *C*–*D*, each through a concerted pair of N—H⋯N hydrogen bonds with a graph-set notation of 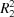
(8), in a similar arrangement to the dimeric structure found in form 1. Figs. 1 ▸ and 2 ▸ show displacement ellipsoid plots for the two dimers, *A*–*B* and *C*–*D* and hydrogen-bond distances and angles are given in Table 1 ▸. The phenyl and pyrimidine rings defined by atoms C1–C6 and N2/N3/C7–C10, respectively, for mol­ecules *A* to *D* are approximately co-planar. A calculated least-squares plane through the six atoms of the phenyl ring and the six atoms of the pyrimidine ring gave r.m.s. deviations from planarity and a calculated dihedral angle between them as follows: mol­ecule *A*, 0.0019 Å, 0.0050 Å, 10.8 (1)°; mol­ecule *B*, 0.0076 Å, 0.0102 Å, 14.8 (1)°; mol­ecule *C*, 0.0049 Å, 0.0153 Å, 8.2 (1)° and mol­ecule *D*, 0.0081 Å, 0.0105 Å, 13.5 (1)°. The small variation in the angular range of the dihedral angles appears consistent with that observed for the other pyrimethanil structures discussed below, 7.5-13.1°.

## Supra­molecular features   

**Figure 3:**
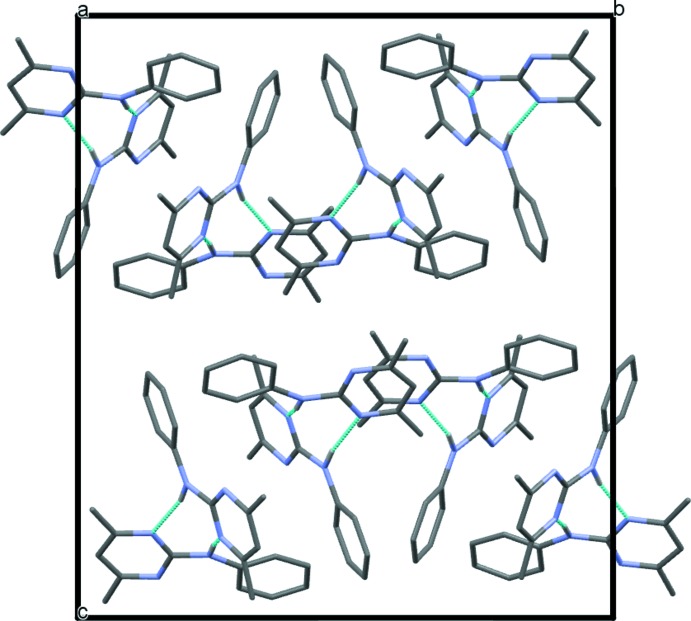
View of the crystal packing down the *a* axis. Only the nitro­gen heteroatom H atoms are shown for clarity. The inter­molecular N—H⋯N hydrogen bonds (see Table 1 ▸) are shown as dotted lines.

**Figure 4:**
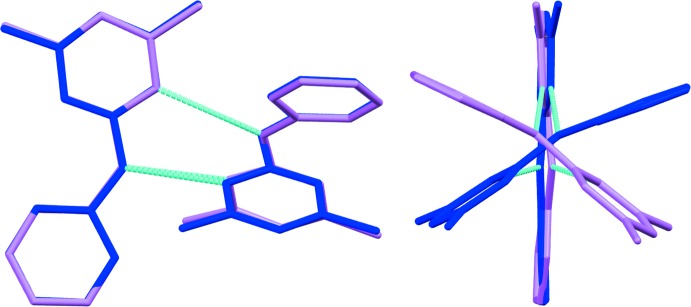
View of the overlay of dimer *A*–*B* (violet) and dimer *C*–*D* (blue).

A view of the crystal packing down the *a*-axis is shown in Fig. 3 ▸. The 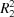
(8) hydrogen-bonded rings defined by atoms N3*A*/C7*A*/N*1A*/H1*AB*/N3*B*/C7*B*/N1*B*/H1*BB* and N3*C*/C7*C*/N1*C*/H1*CB*/N3*D*/C7*D*/N1*D*/H1*DB* for the two dimers are twisted such that each dimer forms a cross pattern, with a dihedral angle of 42.8 (2)° for dimer *A*–*B* and 47.5 (2)° for dimer *C*–*D*. These dihedral angles are between planes C6*A*/N1*A*/C7*A* and C6*B*/N1*B*/C7*B* for *A*–*B* and C6*C*/N1*C*/C7*C* and C6*D*/N1*D*/C7*D* for *C*–*D*. The angles are somewhat reduced in magnitude when compared to the equivalent calculation performed for form 1, 55.7 (1)°. Fig. 4 ▸ shows an overlay of the two dimeric units in form 2, dimer *A*–*B* is shown in violet and *C*–*D* in blue, which reveals the origin of the polymorphic behaviour and in turn the reason why *Z′* = 4. In this figure, mol­ecules *A* and *C* have been overlaid (r.m.s. deviation = 0.181Å) using the standard routine in *Mercury* (Macrae *et al.*, 2008 ▸). It can be seen that mol­ecule *B* in the *A*–*B* dimer is rotated 134° with respect to mol­ecule *D* in the *C*–*D* dimer, thus making each dimer unique. It is inter­esting to note that the dimer found in the structure of form 1 has a similar conformation/orientation to the *C*–*D* dimer in the present structure. There are no further significant inter­molecular contacts and the crystal packing between dimers appears to be driven largely by van der Waals forces only.

## Database survey   

A search of the Cambridge Structural Database (CSD, Version 5.38 update February 2017; Groom *et al.*, 2016 ▸) for both the pyrimethanil framework and its protonated counterpart yielded three hits, all of which were genuine examples of the material under investigation. Only one entry was found which related to an example that was not a co-crystal, solvate or salt form and that was for the triclinic, *P* 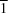, Z′ = 2, form 1 polymorph (CELNOY; Sun *et al.*, 2011 ▸. The remaining two entries were salt forms where the basic nitro­gen atom (N3) had been protonated. These examples are the mono­chloro­acetate (MIRYOC; Li *et al.*, 2008 ▸) and the *p*-toluene­sulfonate (XEZFUE; Li *et al.*, 2007 ▸). One further example, which is not yet available in the current release of the database, is an exciting 1:1 co-crystal of pyrimethanil with a second anti­fungal active, di­thia­non (SAJJAR; Pöppler *et al.*, 2017 ▸). This material is currently being marketed under the trade name FABAN^®^.

## Synthesis and crystallization   

Crystals of form 2 of pyrimethanil were isolated from the reaction product of an attempted co-crystallization screen with *meso*-erythriol in di­methyl­sulfoxide (DMSO). The screen consisted of approximately 20 mg of pyrimethanil being dispensed per vial along with 20 volumes of the appropriate solvent, approx. 400 µl, at room temperature. The appropriate coformer (ratio 1:1) was also dispensed into the vials in the same manner along with a further 20 volumes of solvent. For the vials that gave clear solutions, these were filtered through a 4 µm filter to remove any potential seeds that may remain in the solution. The vials were placed in a platform shaker incubator (Heidolph Titramax/Inkubator 1000) and subjected to a series of heating–cooling cycles under shaking from room temperature (RT) to 323 K (8 h cycles; heating to 323 K for 4 h and then cooling to RT for a further 4 h) for a maximum of 48 h. The resulting solutions were then allowed to evaporate slowly over a period of 14 days. The solid materials obtained from the screen were analysed by X-ray powder diffraction and were investigated further if they displayed diffraction patterns that were clearly different from that of form 1 or the coformer itself. Unfortunately, it has not been possible thus far to repeat the above experiment to generate more form 2 material, leading us to conclude that form 2 is a metastable form with respect to form 1.

## Refinement   

Crystal data, data collection, and structure refinement details are summarized in Table 2 ▸. The positional coordinates of the N-bound H atoms were all located from a Fourier-difference map and freely refined. All the remaining H atoms were placed geometrically in idealized positions and refined using a riding model (including free rotation about the methyl C—C bond), with C—H = 0.95–0.99 Å and *U*_iso_ = 1.5*U*_eq_(C) for methyl groups and 1.2*U*_eq_(C) for other H atoms.

## Supplementary Material

Crystal structure: contains datablock(s) I. DOI: 10.1107/S2056989017007563/hb7679sup1.cif

Structure factors: contains datablock(s) I. DOI: 10.1107/S2056989017007563/hb7679Isup2.hkl

Supporting information file. DOI: 10.1107/S2056989017007563/hb7679Isup3.cml

CCDC reference: 1549998

Additional supporting information:  crystallographic information; 3D view; checkCIF report

## Figures and Tables

**Figure 2:**
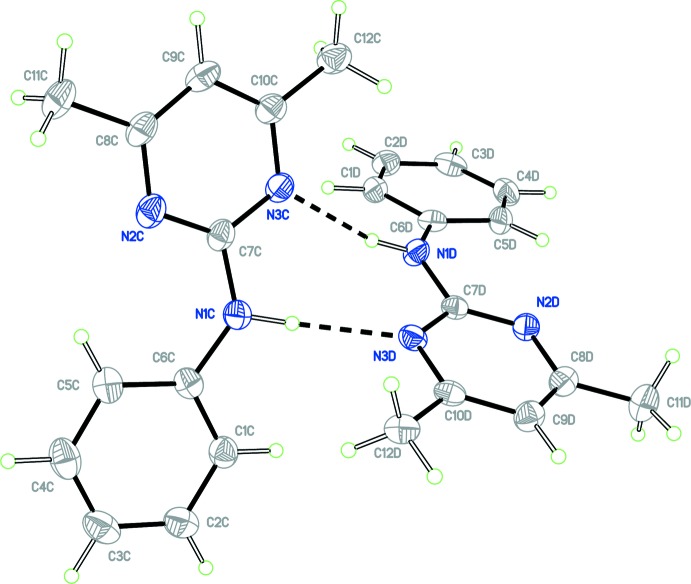
View of the *C*–*D* dimer of the asymmetric unit with atom labelling. Ellipsoids are drawn at the 50% probability level. The inter­molecular N—H⋯N hydrogen bonds are shown as dashed lines.

**Table 1:** Hydrogen-bond geometry (Å, °)

*D*—H⋯*A*	*D*—H	H⋯*A*	*D*⋯*A*	*D*—H⋯*A*
N1*A*—H1*AB*⋯N3*B*	0.91 (3)	2.11 (3)	2.997 (3)	165 (2)
N1*B*—H1*BB*⋯N3*A*	0.97 (3)	2.08 (3)	3.022 (3)	162 (3)
N1*C*—H1*CB*⋯N3*D*	0.94 (3)	2.05 (3)	2.975 (3)	166 (3)
N1*D*—H1*DB*⋯N3*C*	0.92 (3)	2.08 (3)	2.987 (3)	167 (3)

**Table 2:** Experimental details

Crystal data	
Chemical formula	C_12_H_13_N_3_
*M*_r_	199.25
Crystal system, space group	Monoclinic, *P*2_1_/*n*
Temperature (K)	120
*a*, *b*, *c* (Å)	10.5351 (4), 19.1686 (7), 22.1162 (8)
β (°)	102.778 (4)
*V* (Å^3^)	4355.6 (3)
*Z*	16
Radiation type	Mo *K*α
μ (mm^−1^)	0.08
Crystal size (mm)	0.20 × 0.15 × 0.10

Data collection	
Diffractometer	Agilent SuperNova, Dual, Cu at zero, Atlas
Absorption correction	Multi-scan (*CrysAlis PRO*; Rigaku, 2015)
*T*_min_, *T*_max_	0.960, 1.000
No. of measured, independent and observed [*I* > 2σ(*I*)] reflections	16540, 7552, 4410
*R*_int_	0.053
(sin θ/λ)_max_ (Å^−1^)	0.595

Refinement	
*R*[*F* ^2^ > 2σ(*F* ^2^)], *wR*(*F* ^2^), *S*	0.056, 0.149, 1.00
No. of reflections	7552
No. of parameters	565
H-atom treatment	H atoms treated by a mixture of independent and constrained refinement
Δρ_max_, Δρ_min_ (e Å^−3^)	0.24, −0.29

Computer programs: *CrysAlis PRO* (Rigaku, 2015 ▸), *SHELXD2014* (Sheldrick, 2015 ▸), *SHELXL2014* (Sheldrick, 2015 ▸), *SHELXTL* (Sheldrick, 2008 ▸), *Mercury* (Macrae *et al.*, 2008 ▸) and *publCIF* (Westrip, 2010 ▸).

## supplementary crystallographic information

### Crystal data

**Table:**

C_12_H_13_N_3_	*F*(000) = 1696
*M**_r_* = 199.25	*D*_x_ = 1.215 Mg m^−^^3^
Monoclinic, *P*2_1_/*n*	Mo *K*α radiation, λ = 0.71073 Å
*a* = 10.5351 (4) Å	Cell parameters from 2876 reflections
*b* = 19.1686 (7) Å	θ = 2.9–24.5°
*c* = 22.1162 (8) Å	µ = 0.08 mm^−^^1^
β = 102.778 (4)°	*T* = 120 K
*V* = 4355.6 (3) Å^3^	Block, colourless
*Z* = 16	0.20 × 0.15 × 0.10 mm

### Data collection

**Table:**

Agilent SuperNova, Dual, Cu at zero, Atlas diffractometer	7552 independent reflections
Radiation source: SuperNova (Mo) X-ray Source	4410 reflections with *I* > 2σ(*I*)
Mirror monochromator	*R*_int_ = 0.053
Detector resolution: 10.5598 pixels mm^-1^	θ_max_ = 25.0°, θ_min_ = 2.9°
ω scans	*h* = −12→12
Absorption correction: multi-scan (CrysAlis PRO; Rigaku, 2015)	*k* = −22→18
*T*_min_ = 0.960, *T*_max_ = 1.000	*l* = −25→26
16540 measured reflections	

### Refinement

**Table:**

Refinement on *F*^2^	Primary atom site location: structure-invariant direct methods
Least-squares matrix: full	Hydrogen site location: mixed
*R*[*F*^2^ > 2σ(*F*^2^)] = 0.056	H atoms treated by a mixture of independent and constrained refinement
*wR*(*F*^2^) = 0.149	*w* = 1/[σ^2^(*F*_o_^2^) + (0.055*P*)^2^] where *P* = (*F*_o_^2^ + 2*F*_c_^2^)/3
*S* = 1.00	(Δ/σ)_max_ = 0.002
7552 reflections	Δρ_max_ = 0.24 e Å^−^^3^
565 parameters	Δρ_min_ = −0.29 e Å^−^^3^
0 restraints	

### Special details

**Table:**

Geometry. All esds (except the esd in the dihedral angle between two l.s. planes) are estimated using the full covariance matrix. The cell esds are taken into account individually in the estimation of esds in distances, angles and torsion angles; correlations between esds in cell parameters are only used when they are defined by crystal symmetry. An approximate (isotropic) treatment of cell esds is used for estimating esds involving l.s. planes.

### Fractional atomic coordinates and isotropic or equivalent isotropic displacement parameters (Å^2^)

**Table:**

	*x*	*y*	*z*	*U*_iso_*/*U*_eq_	
N1A	0.6124 (2)	0.75456 (11)	0.10435 (11)	0.0263 (6)	
H1AB	0.698 (3)	0.7460 (13)	0.1205 (12)	0.025 (7)*	
N2A	0.4761 (2)	0.84979 (12)	0.06807 (10)	0.0261 (6)	
N3A	0.6823 (2)	0.86154 (11)	0.14057 (10)	0.0256 (5)	
C1A	0.5890 (3)	0.63127 (14)	0.10191 (13)	0.0280 (7)	
H1AA	0.6763	0.6288	0.1251	0.034*	
C2A	0.5192 (3)	0.57049 (15)	0.08619 (13)	0.0328 (7)	
H2AA	0.5586	0.5267	0.0988	0.039*	
C3A	0.3916 (3)	0.57303 (15)	0.05197 (12)	0.0308 (7)	
H3AA	0.3431	0.5314	0.0410	0.037*	
C4A	0.3367 (3)	0.63763 (15)	0.03413 (12)	0.0304 (7)	
H4AA	0.2497	0.6399	0.0106	0.036*	
C5A	0.4056 (2)	0.69886 (15)	0.04986 (12)	0.0276 (7)	
H5AA	0.3659	0.7425	0.0372	0.033*	
C6A	0.5331 (2)	0.69632 (14)	0.08422 (12)	0.0234 (6)	
C7A	0.5858 (3)	0.82446 (14)	0.10375 (13)	0.0245 (7)	
C8A	0.4627 (3)	0.91995 (15)	0.06878 (13)	0.0289 (7)	
C9A	0.5570 (3)	0.96202 (15)	0.10415 (13)	0.0309 (7)	
H9AA	0.5466	1.0113	0.1040	0.037*	
C10A	0.6671 (3)	0.93098 (14)	0.13983 (13)	0.0286 (7)	
C11A	0.3416 (3)	0.94925 (16)	0.02772 (14)	0.0381 (8)	
H11A	0.2651	0.9256	0.0364	0.057*	
H11B	0.3446	0.9420	−0.0158	0.057*	
H11C	0.3361	0.9993	0.0358	0.057*	
C12A	0.7746 (3)	0.97312 (15)	0.17935 (14)	0.0375 (8)	
H12A	0.8564	0.9470	0.1856	0.056*	
H12B	0.7536	0.9824	0.2196	0.056*	
H12C	0.7838	1.0174	0.1586	0.056*	
N1B	0.9217 (2)	0.79655 (12)	0.22174 (11)	0.0244 (5)	
H1BB	0.837 (3)	0.8075 (16)	0.1961 (14)	0.050 (9)*	
N2B	1.0990 (2)	0.72567 (11)	0.21211 (10)	0.0216 (5)	
N3B	0.9013 (2)	0.73288 (11)	0.13366 (10)	0.0232 (5)	
C1B	0.8653 (3)	0.85002 (14)	0.30962 (13)	0.0295 (7)	
H1BA	0.7786	0.8535	0.2858	0.035*	
C2B	0.8942 (3)	0.87347 (14)	0.36999 (13)	0.0316 (7)	
H2BA	0.8275	0.8927	0.3876	0.038*	
C3B	1.0206 (3)	0.86906 (14)	0.40526 (14)	0.0326 (7)	
H3BA	1.0406	0.8848	0.4470	0.039*	
C4B	1.1167 (3)	0.84163 (14)	0.37901 (13)	0.0299 (7)	
H4BA	1.2033	0.8386	0.4030	0.036*	
C5B	1.0887 (3)	0.81842 (14)	0.31799 (13)	0.0261 (7)	
H5BA	1.1563	0.8007	0.3002	0.031*	
C6B	0.9619 (2)	0.82115 (13)	0.28306 (12)	0.0222 (6)	
C7B	0.9786 (2)	0.74942 (14)	0.18899 (12)	0.0220 (6)	
C8B	1.1475 (2)	0.68135 (14)	0.17509 (12)	0.0229 (6)	
C9B	1.0768 (3)	0.66375 (14)	0.11680 (13)	0.0247 (7)	
H9BA	1.1128	0.6339	0.0907	0.030*	
C10B	0.9522 (3)	0.69043 (14)	0.09717 (12)	0.0233 (6)	
C11B	1.2817 (2)	0.65336 (15)	0.20046 (13)	0.0304 (7)	
H11D	1.3404	0.6919	0.2169	0.046*	
H11E	1.3134	0.6298	0.1673	0.046*	
H11F	1.2791	0.6201	0.2338	0.046*	
C12B	0.8698 (3)	0.67444 (15)	0.03397 (12)	0.0307 (7)	
H12D	0.7828	0.6944	0.0303	0.046*	
H12E	0.8627	0.6238	0.0283	0.046*	
H12F	0.9103	0.6947	0.0022	0.046*	
N1C	1.1500 (2)	1.08100 (12)	0.13645 (11)	0.0274 (6)	
H1CB	1.239 (3)	1.0913 (16)	0.1519 (15)	0.058 (10)*	
N2C	1.0238 (2)	0.98550 (12)	0.09055 (10)	0.0285 (6)	
N3C	1.2273 (2)	0.97203 (12)	0.16432 (10)	0.0254 (5)	
C1C	1.1158 (3)	1.20305 (14)	0.13836 (12)	0.0280 (7)	
H1CA	1.2015	1.2066	0.1632	0.034*	
C2C	1.0416 (3)	1.26280 (15)	0.12328 (13)	0.0333 (7)	
H2CA	1.0768	1.3069	0.1377	0.040*	
C3C	0.9166 (3)	1.25855 (16)	0.08734 (13)	0.0360 (8)	
H3CA	0.8647	1.2993	0.0776	0.043*	
C4C	0.8683 (3)	1.19384 (17)	0.06585 (13)	0.0357 (8)	
H4CA	0.7833	1.1907	0.0402	0.043*	
C5C	0.9410 (3)	1.13327 (16)	0.08091 (13)	0.0314 (7)	
H5CA	0.9056	1.0892	0.0664	0.038*	
C6C	1.0665 (3)	1.13804 (14)	0.11756 (12)	0.0247 (7)	
C7C	1.1299 (3)	1.01019 (14)	0.12912 (13)	0.0252 (7)	
C8C	1.0168 (3)	0.91572 (15)	0.08390 (13)	0.0284 (7)	
C9C	1.1147 (3)	0.87277 (15)	0.11533 (13)	0.0302 (7)	
H9CA	1.1106	0.8238	0.1089	0.036*	
C10C	1.2192 (3)	0.90258 (14)	0.15653 (13)	0.0269 (7)	
C11C	0.8976 (3)	0.88820 (16)	0.04020 (13)	0.0352 (8)	
H11G	0.8728	0.9200	0.0048	0.053*	
H11H	0.8261	0.8847	0.0618	0.053*	
H11I	0.9161	0.8419	0.0254	0.053*	
C12C	1.3266 (3)	0.85994 (15)	0.19480 (13)	0.0332 (7)	
H12G	1.4079	0.8867	0.2023	0.050*	
H12H	1.3372	0.8168	0.1726	0.050*	
H12I	1.3049	0.8484	0.2345	0.050*	
N1D	1.4540 (2)	1.03911 (12)	0.24960 (11)	0.0242 (5)	
H1DB	1.378 (3)	1.0251 (15)	0.2234 (13)	0.039 (9)*	
N2D	1.6334 (2)	1.10956 (11)	0.24278 (10)	0.0240 (5)	
N3D	1.4352 (2)	1.10768 (11)	0.16432 (10)	0.0227 (5)	
C1D	1.3924 (3)	0.98318 (14)	0.33508 (13)	0.0276 (7)	
H1DA	1.3072	0.9788	0.3099	0.033*	
C2D	1.4181 (3)	0.95921 (14)	0.39511 (13)	0.0290 (7)	
H2DA	1.3501	0.9391	0.4112	0.035*	
C3D	1.5421 (3)	0.96405 (14)	0.43236 (13)	0.0304 (7)	
H3DA	1.5596	0.9478	0.4739	0.036*	
C4D	1.6398 (3)	0.99302 (14)	0.40792 (13)	0.0285 (7)	
H4DA	1.7253	0.9961	0.4331	0.034*	
C5D	1.6162 (2)	1.01761 (14)	0.34767 (12)	0.0252 (7)	
H5DA	1.6850	1.0368	0.3315	0.030*	
C6D	1.4905 (3)	1.01402 (13)	0.31083 (12)	0.0227 (6)	
C7D	1.5119 (2)	1.08765 (13)	0.21896 (12)	0.0211 (6)	
C8D	1.6839 (3)	1.15457 (14)	0.20693 (13)	0.0259 (7)	
C9D	1.6144 (3)	1.17560 (14)	0.14969 (13)	0.0264 (7)	
H9DA	1.6521	1.2059	0.1246	0.032*	
C10D	1.4874 (3)	1.15146 (13)	0.12938 (12)	0.0227 (6)	
C11D	1.8201 (3)	1.17911 (16)	0.23313 (14)	0.0356 (8)	
H11J	1.8746	1.1393	0.2503	0.053*	
H11K	1.8195	1.2133	0.2660	0.053*	
H11L	1.8553	1.2008	0.2002	0.053*	
C12D	1.4050 (3)	1.17113 (14)	0.06759 (12)	0.0280 (7)	
H12J	1.3215	1.1895	0.0731	0.042*	
H12K	1.3897	1.1299	0.0408	0.042*	
H12L	1.4499	1.2069	0.0484	0.042*	

### Atomic displacement parameters (Å^2^)

**Table:**

	*U*^11^	*U*^22^	*U*^33^	*U*^12^	*U*^13^	*U*^23^
N1A	0.0219 (14)	0.0189 (13)	0.0347 (15)	0.0032 (11)	−0.0007 (12)	−0.0004 (11)
N2A	0.0253 (13)	0.0271 (14)	0.0271 (14)	0.0056 (10)	0.0085 (12)	0.0052 (11)
N3A	0.0276 (13)	0.0204 (13)	0.0298 (14)	0.0016 (10)	0.0081 (12)	−0.0037 (11)
C1A	0.0240 (15)	0.0266 (16)	0.0311 (17)	0.0024 (13)	0.0011 (14)	0.0006 (14)
C2A	0.0337 (18)	0.0251 (16)	0.0398 (19)	0.0014 (14)	0.0085 (16)	−0.0032 (14)
C3A	0.0364 (18)	0.0294 (17)	0.0262 (17)	−0.0101 (14)	0.0059 (15)	−0.0022 (14)
C4A	0.0252 (16)	0.0427 (19)	0.0214 (16)	−0.0045 (14)	0.0009 (14)	0.0023 (14)
C5A	0.0265 (16)	0.0295 (17)	0.0251 (17)	0.0034 (13)	0.0021 (14)	0.0050 (13)
C6A	0.0224 (15)	0.0254 (16)	0.0221 (16)	0.0013 (12)	0.0041 (13)	−0.0002 (13)
C7A	0.0283 (16)	0.0245 (16)	0.0232 (17)	0.0037 (13)	0.0110 (14)	0.0022 (13)
C8A	0.0303 (16)	0.0313 (18)	0.0291 (18)	0.0097 (14)	0.0152 (15)	0.0083 (14)
C9A	0.0382 (18)	0.0221 (16)	0.0357 (19)	0.0080 (14)	0.0150 (16)	0.0023 (14)
C10A	0.0340 (16)	0.0251 (16)	0.0310 (18)	0.0019 (14)	0.0164 (15)	−0.0032 (14)
C11A	0.0343 (17)	0.0355 (18)	0.044 (2)	0.0104 (14)	0.0087 (16)	0.0124 (16)
C12A	0.0437 (19)	0.0260 (17)	0.043 (2)	−0.0022 (14)	0.0105 (17)	−0.0082 (15)
N1B	0.0214 (13)	0.0265 (13)	0.0235 (14)	0.0034 (11)	0.0011 (12)	−0.0061 (11)
N2B	0.0212 (12)	0.0215 (12)	0.0229 (13)	0.0011 (10)	0.0062 (11)	0.0006 (10)
N3B	0.0262 (12)	0.0225 (13)	0.0203 (13)	−0.0026 (10)	0.0038 (11)	−0.0029 (11)
C1B	0.0302 (16)	0.0261 (16)	0.0338 (18)	0.0027 (13)	0.0105 (15)	−0.0036 (14)
C2B	0.0387 (18)	0.0273 (17)	0.0317 (19)	0.0012 (14)	0.0138 (16)	−0.0061 (14)
C3B	0.046 (2)	0.0250 (16)	0.0273 (17)	−0.0012 (14)	0.0084 (16)	−0.0056 (14)
C4B	0.0303 (16)	0.0299 (17)	0.0280 (18)	−0.0013 (13)	0.0034 (14)	−0.0032 (14)
C5B	0.0274 (16)	0.0236 (15)	0.0281 (17)	0.0008 (13)	0.0081 (14)	−0.0024 (13)
C6B	0.0251 (16)	0.0171 (14)	0.0234 (16)	−0.0022 (12)	0.0030 (14)	−0.0005 (12)
C7B	0.0221 (15)	0.0205 (14)	0.0240 (16)	−0.0028 (12)	0.0062 (14)	0.0005 (13)
C8B	0.0239 (15)	0.0225 (15)	0.0233 (16)	0.0022 (12)	0.0076 (14)	0.0053 (13)
C9B	0.0284 (16)	0.0231 (15)	0.0250 (17)	0.0006 (12)	0.0109 (14)	−0.0038 (13)
C10B	0.0288 (15)	0.0207 (15)	0.0212 (16)	−0.0027 (12)	0.0073 (14)	0.0024 (13)
C11B	0.0278 (16)	0.0327 (17)	0.0294 (17)	0.0072 (13)	0.0032 (14)	−0.0010 (14)
C12B	0.0320 (16)	0.0324 (17)	0.0267 (17)	0.0003 (14)	0.0043 (14)	−0.0022 (14)
N1C	0.0240 (14)	0.0253 (14)	0.0301 (15)	−0.0001 (11)	−0.0002 (12)	−0.0008 (11)
N2C	0.0263 (13)	0.0323 (14)	0.0247 (14)	−0.0054 (11)	0.0005 (12)	0.0002 (11)
N3C	0.0243 (13)	0.0256 (14)	0.0248 (14)	−0.0040 (10)	0.0022 (11)	0.0030 (11)
C1C	0.0257 (15)	0.0320 (17)	0.0253 (17)	−0.0002 (13)	0.0035 (14)	0.0034 (14)
C2C	0.0367 (18)	0.0299 (17)	0.0337 (18)	0.0038 (14)	0.0086 (16)	0.0043 (15)
C3C	0.0395 (19)	0.0359 (19)	0.0348 (19)	0.0111 (15)	0.0130 (17)	0.0109 (15)
C4C	0.0289 (17)	0.051 (2)	0.0263 (18)	0.0053 (15)	0.0037 (15)	0.0039 (16)
C5C	0.0263 (16)	0.0366 (18)	0.0310 (18)	0.0012 (14)	0.0057 (15)	−0.0008 (14)
C6C	0.0254 (16)	0.0293 (17)	0.0195 (16)	0.0028 (13)	0.0055 (14)	0.0032 (13)
C7C	0.0232 (16)	0.0297 (17)	0.0218 (16)	−0.0043 (13)	0.0030 (14)	0.0020 (13)
C8C	0.0285 (16)	0.0322 (18)	0.0250 (17)	−0.0099 (14)	0.0071 (14)	0.0019 (14)
C9C	0.0362 (17)	0.0253 (16)	0.0283 (17)	−0.0101 (14)	0.0056 (15)	0.0017 (14)
C10C	0.0293 (16)	0.0268 (17)	0.0254 (17)	−0.0035 (13)	0.0079 (14)	0.0033 (13)
C11C	0.0334 (17)	0.0402 (19)	0.0289 (17)	−0.0138 (14)	0.0003 (15)	−0.0041 (15)
C12C	0.0367 (17)	0.0281 (17)	0.0327 (18)	−0.0041 (14)	0.0033 (15)	0.0062 (14)
N1D	0.0219 (13)	0.0243 (13)	0.0228 (14)	−0.0046 (11)	−0.0032 (12)	0.0054 (11)
N2D	0.0226 (13)	0.0231 (13)	0.0264 (13)	−0.0004 (10)	0.0059 (11)	−0.0020 (11)
N3D	0.0238 (12)	0.0205 (12)	0.0235 (13)	0.0034 (10)	0.0048 (11)	0.0039 (11)
C1D	0.0222 (15)	0.0250 (16)	0.0329 (18)	−0.0002 (12)	0.0006 (14)	0.0049 (14)
C2D	0.0318 (17)	0.0255 (16)	0.0310 (18)	−0.0005 (13)	0.0095 (15)	0.0070 (14)
C3D	0.0424 (19)	0.0229 (16)	0.0258 (17)	0.0080 (14)	0.0076 (16)	0.0027 (13)
C4D	0.0258 (16)	0.0296 (17)	0.0278 (17)	0.0061 (13)	0.0007 (14)	−0.0003 (14)
C5D	0.0201 (15)	0.0281 (16)	0.0256 (17)	−0.0009 (12)	0.0012 (14)	−0.0007 (13)
C6D	0.0303 (16)	0.0163 (14)	0.0211 (16)	0.0021 (12)	0.0052 (14)	−0.0001 (12)
C7D	0.0229 (15)	0.0171 (14)	0.0238 (16)	0.0021 (12)	0.0059 (14)	−0.0031 (13)
C8D	0.0270 (15)	0.0265 (16)	0.0254 (17)	−0.0021 (13)	0.0083 (14)	−0.0026 (14)
C9D	0.0288 (16)	0.0274 (16)	0.0259 (17)	−0.0020 (13)	0.0119 (15)	0.0000 (13)
C10D	0.0289 (16)	0.0177 (14)	0.0222 (16)	0.0043 (12)	0.0072 (14)	−0.0033 (12)
C11D	0.0291 (16)	0.0438 (19)	0.0348 (19)	−0.0069 (14)	0.0089 (15)	0.0015 (15)
C12D	0.0337 (16)	0.0231 (16)	0.0269 (17)	0.0004 (13)	0.0059 (14)	0.0012 (13)

### Geometric parameters (Å, º)

**Table:**

N1A—C7A	1.368 (3)	N1C—C7C	1.378 (3)
N1A—C6A	1.407 (3)	N1C—C6C	1.408 (3)
N1A—H1AB	0.91 (3)	N1C—H1CB	0.94 (3)
N2A—C7A	1.338 (3)	N2C—C7C	1.334 (3)
N2A—C8A	1.353 (3)	N2C—C8C	1.346 (3)
N3A—C10A	1.340 (3)	N3C—C10C	1.343 (3)
N3A—C7A	1.354 (3)	N3C—C7C	1.357 (3)
C1A—C2A	1.380 (4)	C1C—C2C	1.385 (4)
C1A—C6A	1.398 (4)	C1C—C6C	1.389 (4)
C1A—H1AA	0.9500	C1C—H1CA	0.9500
C2A—C3A	1.390 (4)	C2C—C3C	1.382 (4)
C2A—H2AA	0.9500	C2C—H2CA	0.9500
C3A—C4A	1.387 (4)	C3C—C4C	1.384 (4)
C3A—H3AA	0.9500	C3C—H3CA	0.9500
C4A—C5A	1.383 (4)	C4C—C5C	1.391 (4)
C4A—H4AA	0.9500	C4C—H4CA	0.9500
C5A—C6A	1.390 (4)	C5C—C6C	1.392 (4)
C5A—H5AA	0.9500	C5C—H5CA	0.9500
C8A—C9A	1.380 (4)	C8C—C9C	1.382 (4)
C8A—C11A	1.501 (4)	C8C—C11C	1.501 (4)
C9A—C10A	1.385 (4)	C9C—C10C	1.387 (4)
C9A—H9AA	0.9500	C9C—H9CA	0.9500
C10A—C12A	1.504 (4)	C10C—C12C	1.497 (4)
C11A—H11A	0.9800	C11C—H11G	0.9800
C11A—H11B	0.9800	C11C—H11H	0.9800
C11A—H11C	0.9800	C11C—H11I	0.9800
C12A—H12A	0.9800	C12C—H12G	0.9800
C12A—H12B	0.9800	C12C—H12H	0.9800
C12A—H12C	0.9800	C12C—H12I	0.9800
N1B—C7B	1.375 (3)	N1D—C7D	1.371 (3)
N1B—C6B	1.410 (3)	N1D—C6D	1.408 (3)
N1B—H1BB	0.97 (3)	N1D—H1DB	0.92 (3)
N2B—C7B	1.338 (3)	N2D—C7D	1.339 (3)
N2B—C8B	1.356 (3)	N2D—C8D	1.357 (3)
N3B—C10B	1.339 (3)	N3D—C10D	1.339 (3)
N3B—C7B	1.349 (3)	N3D—C7D	1.352 (3)
C1B—C2B	1.378 (4)	C1D—C2D	1.374 (4)
C1B—C6B	1.397 (3)	C1D—C6D	1.397 (3)
C1B—H1BA	0.9500	C1D—H1DA	0.9500
C2B—C3B	1.389 (4)	C2D—C3D	1.384 (4)
C2B—H2BA	0.9500	C2D—H2DA	0.9500
C3B—C4B	1.378 (4)	C3D—C4D	1.380 (4)
C3B—H3BA	0.9500	C3D—H3DA	0.9500
C4B—C5B	1.389 (4)	C4D—C5D	1.383 (4)
C4B—H4BA	0.9500	C4D—H4DA	0.9500
C5B—C6B	1.388 (4)	C5D—C6D	1.394 (4)
C5B—H5BA	0.9500	C5D—H5DA	0.9500
C8B—C9B	1.381 (4)	C8D—C9D	1.375 (4)
C8B—C11B	1.500 (4)	C8D—C11D	1.500 (4)
C9B—C10B	1.386 (4)	C9D—C10D	1.392 (4)
C9B—H9BA	0.9500	C9D—H9DA	0.9500
C10B—C12B	1.505 (4)	C10D—C12D	1.496 (4)
C11B—H11D	0.9800	C11D—H11J	0.9800
C11B—H11E	0.9800	C11D—H11K	0.9800
C11B—H11F	0.9800	C11D—H11L	0.9800
C12B—H12D	0.9800	C12D—H12J	0.9800
C12B—H12E	0.9800	C12D—H12K	0.9800
C12B—H12F	0.9800	C12D—H12L	0.9800
			
C7A—N1A—C6A	131.9 (2)	C7C—N1C—C6C	131.4 (3)
C7A—N1A—H1AB	111.3 (17)	C7C—N1C—H1CB	111 (2)
C6A—N1A—H1AB	116.8 (16)	C6C—N1C—H1CB	117 (2)
C7A—N2A—C8A	115.7 (3)	C7C—N2C—C8C	116.1 (3)
C10A—N3A—C7A	116.2 (3)	C10C—N3C—C7C	116.3 (2)
C2A—C1A—C6A	121.0 (3)	C2C—C1C—C6C	120.8 (3)
C2A—C1A—H1AA	119.5	C2C—C1C—H1CA	119.6
C6A—C1A—H1AA	119.5	C6C—C1C—H1CA	119.6
C1A—C2A—C3A	120.3 (3)	C3C—C2C—C1C	120.3 (3)
C1A—C2A—H2AA	119.8	C3C—C2C—H2CA	119.8
C3A—C2A—H2AA	119.8	C1C—C2C—H2CA	119.8
C4A—C3A—C2A	118.6 (3)	C2C—C3C—C4C	118.8 (3)
C4A—C3A—H3AA	120.7	C2C—C3C—H3CA	120.6
C2A—C3A—H3AA	120.7	C4C—C3C—H3CA	120.6
C5A—C4A—C3A	121.6 (3)	C3C—C4C—C5C	121.6 (3)
C5A—C4A—H4AA	119.2	C3C—C4C—H4CA	119.2
C3A—C4A—H4AA	119.2	C5C—C4C—H4CA	119.2
C4A—C5A—C6A	119.8 (3)	C4C—C5C—C6C	119.1 (3)
C4A—C5A—H5AA	120.1	C4C—C5C—H5CA	120.4
C6A—C5A—H5AA	120.1	C6C—C5C—H5CA	120.4
C5A—C6A—C1A	118.7 (3)	C1C—C6C—C5C	119.3 (3)
C5A—C6A—N1A	125.4 (3)	C1C—C6C—N1C	115.8 (2)
C1A—C6A—N1A	115.8 (2)	C5C—C6C—N1C	124.9 (3)
N2A—C7A—N3A	126.8 (2)	N2C—C7C—N3C	126.6 (3)
N2A—C7A—N1A	120.6 (3)	N2C—C7C—N1C	120.7 (3)
N3A—C7A—N1A	112.5 (2)	N3C—C7C—N1C	112.7 (2)
N2A—C8A—C9A	121.5 (3)	N2C—C8C—C9C	121.3 (3)
N2A—C8A—C11A	116.2 (3)	N2C—C8C—C11C	116.0 (3)
C9A—C8A—C11A	122.2 (3)	C9C—C8C—C11C	122.7 (3)
C8A—C9A—C10A	118.6 (3)	C8C—C9C—C10C	118.7 (3)
C8A—C9A—H9AA	120.7	C8C—C9C—H9CA	120.6
C10A—C9A—H9AA	120.7	C10C—C9C—H9CA	120.6
N3A—C10A—C9A	121.1 (3)	N3C—C10C—C9C	120.8 (3)
N3A—C10A—C12A	117.0 (3)	N3C—C10C—C12C	116.8 (3)
C9A—C10A—C12A	121.9 (3)	C9C—C10C—C12C	122.5 (3)
C8A—C11A—H11A	109.5	C8C—C11C—H11G	109.5
C8A—C11A—H11B	109.5	C8C—C11C—H11H	109.5
H11A—C11A—H11B	109.5	H11G—C11C—H11H	109.5
C8A—C11A—H11C	109.5	C8C—C11C—H11I	109.5
H11A—C11A—H11C	109.5	H11G—C11C—H11I	109.5
H11B—C11A—H11C	109.5	H11H—C11C—H11I	109.5
C10A—C12A—H12A	109.5	C10C—C12C—H12G	109.5
C10A—C12A—H12B	109.5	C10C—C12C—H12H	109.5
H12A—C12A—H12B	109.5	H12G—C12C—H12H	109.5
C10A—C12A—H12C	109.5	C10C—C12C—H12I	109.5
H12A—C12A—H12C	109.5	H12G—C12C—H12I	109.5
H12B—C12A—H12C	109.5	H12H—C12C—H12I	109.5
C7B—N1B—C6B	130.8 (2)	C7D—N1D—C6D	130.6 (3)
C7B—N1B—H1BB	106.7 (18)	C7D—N1D—H1DB	108.0 (17)
C6B—N1B—H1BB	122.1 (17)	C6D—N1D—H1DB	121.4 (17)
C7B—N2B—C8B	115.8 (2)	C7D—N2D—C8D	115.7 (2)
C10B—N3B—C7B	116.6 (2)	C10D—N3D—C7D	116.9 (2)
C2B—C1B—C6B	120.7 (3)	C2D—C1D—C6D	120.5 (3)
C2B—C1B—H1BA	119.6	C2D—C1D—H1DA	119.8
C6B—C1B—H1BA	119.6	C6D—C1D—H1DA	119.8
C1B—C2B—C3B	120.3 (3)	C1D—C2D—C3D	120.8 (3)
C1B—C2B—H2BA	119.9	C1D—C2D—H2DA	119.6
C3B—C2B—H2BA	119.9	C3D—C2D—H2DA	119.6
C4B—C3B—C2B	119.3 (3)	C4D—C3D—C2D	118.7 (3)
C4B—C3B—H3BA	120.4	C4D—C3D—H3DA	120.6
C2B—C3B—H3BA	120.4	C2D—C3D—H3DA	120.6
C3B—C4B—C5B	120.9 (3)	C3D—C4D—C5D	121.6 (3)
C3B—C4B—H4BA	119.5	C3D—C4D—H4DA	119.2
C5B—C4B—H4BA	119.5	C5D—C4D—H4DA	119.2
C6B—C5B—C4B	120.0 (2)	C4D—C5D—C6D	119.4 (2)
C6B—C5B—H5BA	120.0	C4D—C5D—H5DA	120.3
C4B—C5B—H5BA	120.0	C6D—C5D—H5DA	120.3
C5B—C6B—C1B	118.8 (3)	C5D—C6D—C1D	119.0 (2)
C5B—C6B—N1B	124.8 (2)	C5D—C6D—N1D	124.6 (2)
C1B—C6B—N1B	116.4 (2)	C1D—C6D—N1D	116.5 (2)
N2B—C7B—N3B	126.7 (2)	N2D—C7D—N3D	126.3 (2)
N2B—C7B—N1B	120.6 (3)	N2D—C7D—N1D	120.6 (3)
N3B—C7B—N1B	112.7 (2)	N3D—C7D—N1D	113.0 (2)
N2B—C8B—C9B	121.3 (2)	N2D—C8D—C9D	121.8 (2)
N2B—C8B—C11B	116.6 (2)	N2D—C8D—C11D	116.0 (3)
C9B—C8B—C11B	122.1 (2)	C9D—C8D—C11D	122.2 (2)
C8B—C9B—C10B	118.7 (2)	C8D—C9D—C10D	118.5 (2)
C8B—C9B—H9BA	120.6	C8D—C9D—H9DA	120.7
C10B—C9B—H9BA	120.6	C10D—C9D—H9DA	120.7
N3B—C10B—C9B	120.9 (3)	N3D—C10D—C9D	120.7 (3)
N3B—C10B—C12B	117.3 (2)	N3D—C10D—C12D	117.1 (2)
C9B—C10B—C12B	121.8 (2)	C9D—C10D—C12D	122.2 (2)
C8B—C11B—H11D	109.5	C8D—C11D—H11J	109.5
C8B—C11B—H11E	109.5	C8D—C11D—H11K	109.5
H11D—C11B—H11E	109.5	H11J—C11D—H11K	109.5
C8B—C11B—H11F	109.5	C8D—C11D—H11L	109.5
H11D—C11B—H11F	109.5	H11J—C11D—H11L	109.5
H11E—C11B—H11F	109.5	H11K—C11D—H11L	109.5
C10B—C12B—H12D	109.5	C10D—C12D—H12J	109.5
C10B—C12B—H12E	109.5	C10D—C12D—H12K	109.5
H12D—C12B—H12E	109.5	H12J—C12D—H12K	109.5
C10B—C12B—H12F	109.5	C10D—C12D—H12L	109.5
H12D—C12B—H12F	109.5	H12J—C12D—H12L	109.5
H12E—C12B—H12F	109.5	H12K—C12D—H12L	109.5
			
C6A—C1A—C2A—C3A	0.4 (4)	C6C—C1C—C2C—C3C	0.3 (4)
C1A—C2A—C3A—C4A	0.1 (4)	C1C—C2C—C3C—C4C	−1.2 (4)
C2A—C3A—C4A—C5A	−0.3 (4)	C2C—C3C—C4C—C5C	1.7 (4)
C3A—C4A—C5A—C6A	0.2 (4)	C3C—C4C—C5C—C6C	−1.2 (4)
C4A—C5A—C6A—C1A	0.3 (4)	C2C—C1C—C6C—C5C	0.2 (4)
C4A—C5A—C6A—N1A	−178.5 (2)	C2C—C1C—C6C—N1C	−179.2 (2)
C2A—C1A—C6A—C5A	−0.5 (4)	C4C—C5C—C6C—C1C	0.2 (4)
C2A—C1A—C6A—N1A	178.3 (2)	C4C—C5C—C6C—N1C	179.6 (2)
C7A—N1A—C6A—C5A	9.4 (4)	C7C—N1C—C6C—C1C	173.8 (3)
C7A—N1A—C6A—C1A	−169.3 (3)	C7C—N1C—C6C—C5C	−5.6 (4)
C8A—N2A—C7A—N3A	1.0 (4)	C8C—N2C—C7C—N3C	−3.5 (4)
C8A—N2A—C7A—N1A	−176.9 (2)	C8C—N2C—C7C—N1C	176.3 (2)
C10A—N3A—C7A—N2A	−1.8 (4)	C10C—N3C—C7C—N2C	4.3 (4)
C10A—N3A—C7A—N1A	176.3 (2)	C10C—N3C—C7C—N1C	−175.6 (2)
C6A—N1A—C7A—N2A	−16.4 (4)	C6C—N1C—C7C—N2C	12.0 (4)
C6A—N1A—C7A—N3A	165.3 (2)	C6C—N1C—C7C—N3C	−168.1 (2)
C7A—N2A—C8A—C9A	0.2 (3)	C7C—N2C—C8C—C9C	−0.2 (4)
C7A—N2A—C8A—C11A	178.5 (2)	C7C—N2C—C8C—C11C	−179.7 (2)
N2A—C8A—C9A—C10A	−0.4 (4)	N2C—C8C—C9C—C10C	2.9 (4)
C11A—C8A—C9A—C10A	−178.7 (2)	C11C—C8C—C9C—C10C	−177.7 (2)
C7A—N3A—C10A—C9A	1.4 (3)	C7C—N3C—C10C—C9C	−1.2 (3)
C7A—N3A—C10A—C12A	−178.7 (2)	C7C—N3C—C10C—C12C	179.6 (2)
C8A—C9A—C10A—N3A	−0.4 (4)	C8C—C9C—C10C—N3C	−2.1 (4)
C8A—C9A—C10A—C12A	179.7 (2)	C8C—C9C—C10C—C12C	177.1 (2)
C6B—C1B—C2B—C3B	0.4 (4)	C6D—C1D—C2D—C3D	−1.1 (4)
C1B—C2B—C3B—C4B	0.6 (4)	C1D—C2D—C3D—C4D	−0.4 (4)
C2B—C3B—C4B—C5B	−0.1 (4)	C2D—C3D—C4D—C5D	0.6 (4)
C3B—C4B—C5B—C6B	−1.5 (4)	C3D—C4D—C5D—C6D	0.9 (4)
C4B—C5B—C6B—C1B	2.5 (4)	C4D—C5D—C6D—C1D	−2.4 (4)
C4B—C5B—C6B—N1B	−177.4 (2)	C4D—C5D—C6D—N1D	178.1 (2)
C2B—C1B—C6B—C5B	−1.9 (4)	C2D—C1D—C6D—C5D	2.5 (4)
C2B—C1B—C6B—N1B	177.9 (2)	C2D—C1D—C6D—N1D	−177.9 (2)
C7B—N1B—C6B—C5B	20.7 (4)	C7D—N1D—C6D—C5D	−21.4 (4)
C7B—N1B—C6B—C1B	−159.1 (2)	C7D—N1D—C6D—C1D	159.0 (2)
C8B—N2B—C7B—N3B	1.1 (4)	C8D—N2D—C7D—N3D	−2.2 (4)
C8B—N2B—C7B—N1B	−177.7 (2)	C8D—N2D—C7D—N1D	176.3 (2)
C10B—N3B—C7B—N2B	−2.7 (4)	C10D—N3D—C7D—N2D	2.9 (4)
C10B—N3B—C7B—N1B	176.2 (2)	C10D—N3D—C7D—N1D	−175.6 (2)
C6B—N1B—C7B—N2B	−9.0 (4)	C6D—N1D—C7D—N2D	13.2 (4)
C6B—N1B—C7B—N3B	172.0 (2)	C6D—N1D—C7D—N3D	−168.1 (2)
C7B—N2B—C8B—C9B	1.4 (3)	C7D—N2D—C8D—C9D	−0.5 (4)
C7B—N2B—C8B—C11B	−179.2 (2)	C7D—N2D—C8D—C11D	−179.7 (2)
N2B—C8B—C9B—C10B	−2.2 (4)	N2D—C8D—C9D—C10D	2.1 (4)
C11B—C8B—C9B—C10B	178.5 (2)	C11D—C8D—C9D—C10D	−178.7 (2)
C7B—N3B—C10B—C9B	1.8 (3)	C7D—N3D—C10D—C9D	−1.1 (3)
C7B—N3B—C10B—C12B	−176.9 (2)	C7D—N3D—C10D—C12D	177.2 (2)
C8B—C9B—C10B—N3B	0.5 (4)	C8D—C9D—C10D—N3D	−1.3 (4)
C8B—C9B—C10B—C12B	179.1 (2)	C8D—C9D—C10D—C12D	−179.5 (2)

### Hydrogen-bond geometry (Å, º)

**Table:**

*D*—H···*A*	*D*—H	H···*A*	*D*···*A*	*D*—H···*A*
N1*A*—H1*AB*···N3*B*	0.91 (3)	2.11 (3)	2.997 (3)	165 (2)
N1*B*—H1*BB*···N3*A*	0.97 (3)	2.08 (3)	3.022 (3)	162 (3)
N1*C*—H1*CB*···N3*D*	0.94 (3)	2.05 (3)	2.975 (3)	166 (3)
N1*D*—H1*DB*···N3*C*	0.92 (3)	2.08 (3)	2.987 (3)	167 (3)
